# Jan K. Melichar, BSc, MB, BS, MD, FRCPsych

**DOI:** 10.1192/bjb.2025.10189

**Published:** 2026-06

**Authors:** David J. Nutt

Formerly National Health Service consultant addiction psychiatrist, and chair and policy lead of the Faculty of Addictions Psychiatry, Royal College of Psychiatrists Wales

**Figure:**
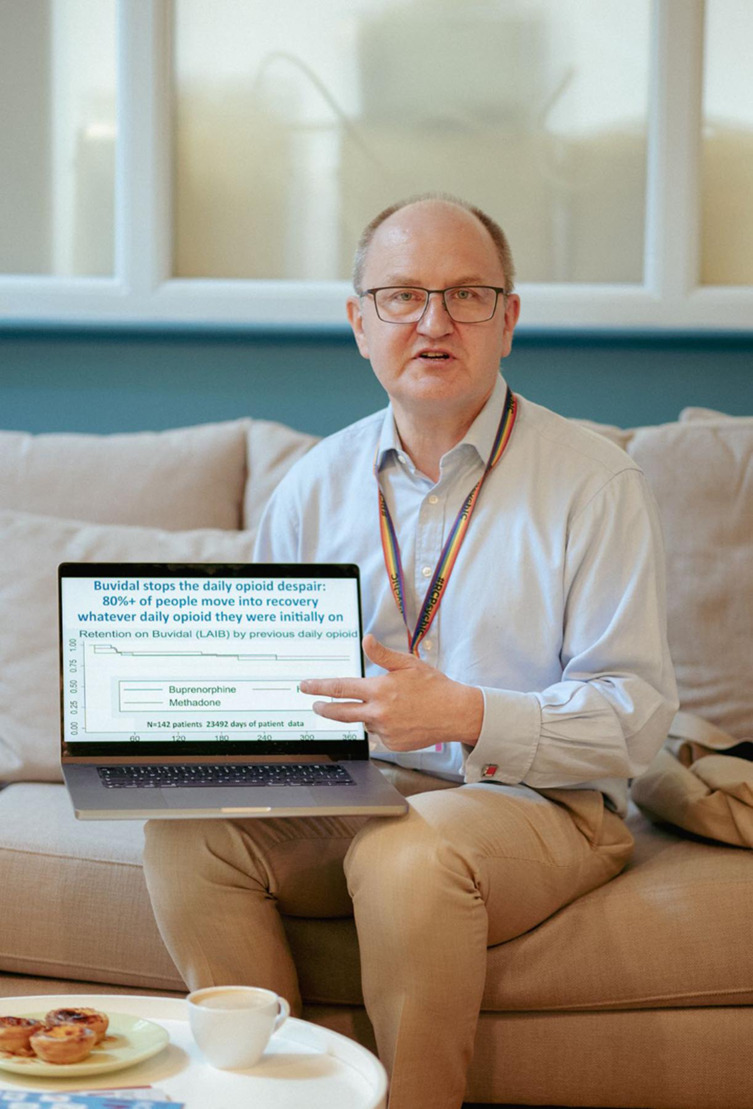

Professor Jan Melichar died suddenly on 31 August 2025 while cycling, aged just 57. Jan was a National Health Service Consultant Addiction Psychiatrist working in Newport and Cardiff, the Faculty Chair of Addictions Psychiatry, for the Royal College of Psychiatrists Wales and its policy lead. He was the UK’s leading expert on the clinical pharmacology of substitution treatments for opioid addiction, especially buprenorphine. For his MD he used positron emission tomography (PET) imaging to conduct the definitive study of human brain pharmacology and then spearheaded the roll-out of long-acting versions of buprenorphine, particularly Buvidal. This proved a hugely effective treatment innovation that supported hundreds of patients in Wales through the COVID-19 pandemic and is now being used much more widely. The success of this programme reflects a rather special combination of Jan’s research training and energetic ‘can do’ approach to problems: he himself gave many hundreds of these injections to patients. His contributions have been recognised by an Honorary Senior Lectureship in Psychopharmacology at the University of Bath and a Visiting Professorship at the University of South Wales.

Jan was born in London on 13 May 1968 to two Polish parents who came to the UK through the resettlement of Polish refugees initiated by Winston Churchill after the Second World War. His father was a teacher and his mother a very socially committed general practitioner. Jan only spoke Polish till he attended infant school but picked up English well enough to get into St Paul’s School, London, which he described to me later as an examination hothouse rather than a place that taught thinking skills. He then graduated in medicine at the Middlesex Hospital before training in psychiatry in the south west, where he also learned to surf and climb rocks!

It was there that I met him in the early 1990s when he attended a lecture given by me in the old Bodmin asylum and afterwards asked if he could do research with my team at Bristol University. I was able to facilitate this and Jan moved to Bristol University to study for his MD, working with Dr Andrea Malizia at the MRC Cyclotron Unit, Imperial College London. There he utilised cutting-edge PET imaging to explore the pharmacology of opioid drugs in the human brain, conducting the definitive studies of brain receptor occupation for the antagonist naloxone, the agonist methadone and, most significantly, the partial agonist buprenorphine. At the time, buprenorphine was being evaluated as a new treatment for opioid dependence as an alternative to methadone. Jan showing that buprenorphine had a very different pharmacology from methadone was a key learning in our understanding of the potential advantages of buprenorphine over methadone. It inspired Jan to move into clinical work to explore this issue. He was part of the Bristol University team that conducted the first transfer of patients from methadone to buprenorphine and the first comparative study of buprenorphine versus methadone in getting ‘street users’ of heroin into treatment and then abstinence. This pioneering work resulted in Jan being awarded a Higher Education Funding Council for England ‘New Blood’ Clinical Senior Lectureship, which initiated two decades of clinical research on different buprenorphine formulations that has resulted in its now widespread use. This work led to Jan being elected a Fellow of the Royal College of Psychiatrists in 2015.

Jan’s research in psychopharmacology extended to novel treatments for opioid withdrawal, such as lofexidine, and he also dealt with the complexities of opioid use in patients with pain disorders when he worked as Medical Director, Substance Misuse Lead in a Regional Complex Pain Service, Southmead Hospital Bristol. He was also the lead consultant in a Regional Psychopharmacology Unit, seeing patients with treatment-resistant depression, anxiety and sleep disorders. Jan also taught on the British Association for Psychopharmacology anxiety psychopharmacology course for nearly 20 years, where his knowledge and clear, no-nonsense approach was well appreciated.

Jan was always innovative and started working with Dr Lucy Donaldson in the dental department at Bristol University to explore if they could use changes in taste to study effects of drugs. As well as revealing massive changes in sweet sensitivity in patients on methadone, which may contribute to its metabolic effects, he found that selective serotonin reuptake inhibitors (SSRIs) such as paroxetine almost immediately profoundly altered taste thresholds. Based on this he co-founded a company called Ranvier Health to develop a simple test system to assess this in patients. This work revealed that early changes in taste sensitivity can predict the effectiveness of an SSRI antidepressant, and larger predictive trials are under development. His research involvement with buprenorphine continued in collaboration with University of Bath medicinal chemists to seek more understanding of the pharmacology of long-acting buprenorphine products and so develop even better treatments.

The success of Jan’s Welsh buprenorphine initiative led to advisory roles both nationally and internationally. He provided expertise to the UK Government, the pharmaceutical industry and health commissioners throughout Europe and the Middle East.

Outside of work, Jan’s enthusiastic personality permeated all aspects of his life. He was an energetic cyclist and skier, a very talented Polish dancer and a dedicated father. He is survived by his second wife, Rosemary, his children, Ania, Thomas and Charlie, plus his stepchildren, Alex and Robert.

